# Efficacy of Periodontal Endoscopy during Subgingival Debridement to Treat Periodontitis: A Systematic Review of Randomized Clinical Trials

**DOI:** 10.3390/dj11050112

**Published:** 2023-04-25

**Authors:** Carlos M. Ardila, Annie Marcela Vivares-Builes

**Affiliations:** 1 Basic Studies Department, School of Dentistry, Universidad de Antioquia UdeA, Medellín 050010, Colombia; 2 School of Dentistry, Institución Universitaria Visión de Las Américas, Medellín 050031, Colombia; anny.vivares@uam.edu.co

**Keywords:** periodontitis, periodontal debridement, endoscopy, systematic review

## Abstract

This study aims to evaluate the clinical efficacy of periodontal endoscopy (PEND) during subgingival debridement to treat periodontitis. A systematic review of randomized clinical trials (RCTs) was performed. The search strategy included four databases: PubMed, Web of Sciences, Scopus, and Scielo. The initial online exploration generated 228 reports, and 3 RCTs met the selection criteria. These RCTs described a statistically significant decrease in probing depth (PD) in the PEND group compared to controls after 6 and 12 months of follow-up. The improvement in PD was 2.5 mm for PEND and 1.8 mm for the control groups, respectively (*p* < 0.05). It was also described that the PEND group presented a significantly inferior proportion of PD 7 to 9 mm at 12 months (0.5%) as compared to the control group (1.84%) (*p* = 0.03). All RCTs noted improvements in clinical attachment level (CAL). It was described as having significant differences in bleeding on probing (BOP) in favor of PEND, with an average reduction of 43% versus 21% in the control groups. Similarly, it was also presented that they were significant differences in plaque indices in favor of PEND. PEND during subgingival debridement to treat periodontitis demonstrated efficacy in reducing PD. Improvement was also observed in CAL and BOP.

## 1. Introduction

Periodontitis is one of the most prevalent chronic infectious diseases in the world. This disease is characterized by the destruction of the supporting tissues of the tooth that can eventually cause tooth loss and results from a complex interplay between the subgingival biofilm and the host response [1]. Thus, host reactions regulate most of the tissue destruction that leads to the clinical expressions of the disease [2]. Plaque biofilm has a primary and gradual role in periodontal disease where mineralization into calculus turns as a holding device for non-calcified plaque, offering ideal settings for microorganisms to colonize and metabolize, thereby hindering periodontal therapy [2,3]. Therefore, successful programs for the timely diagnosis and treatment of periodontitis may be pertinent to restricting the systemic damage that this disease can cause [2].

The essential goal of initial periodontal therapy is the removal of subgingival biofilm and its bacterial products along with calculus elimination to decrease the probing depth and subsequently avoid clinical periodontal attachment loss [3]. Subgingival debridement using hand tools or ultrasound has been shown to be effective in removing bacterial plaque and calculus, decreasing inflammation, and restoring periodontal health. Unfortunately, not all intervened sites present the same satisfactory response, which can be affected by anatomical factors in addition to access and visibility [4]. Therefore, the total elimination of bacterial deposits and calculus is not obtained after debridement, a matter that becomes even more difficult as the depth of the pocket increases [5]. It has been shown that those that have remaining calculus present more insertion loss compared to those teeth that do not; therefore, the removal of these deposits decreases periodontal inflammation, improving the health of the tissues. Precisely because the presence of residual calculus decreases the efficacy of mechanical therapy, possibilities adjunct to conventional treatment arises [6]. Although no clear agreements related to the clinical criteria necessary to guide clinical decisions regarding residual periodontal pockets have been described [7,8], some adjunct therapeutic options have been suggested, including antimicrobial treatment [9,10], surgery [11], laser technology [12], and periodontal endoscopy (PEND) [13], among others.

Endoscope equipment has been adjusted for its implementation in periodontics, specifically for the identification of tissue images. Therefore, PEND allows visualization of the subgingival environment and the position of calculus. This technology permits the subgingival imaging of the periodontal tissues at amplifications of 24 to 48. Subsequently, the obtained image is transmitted to a monitor, offering an immediate visualization of the peri-radicular tissues [6,13].

Subgingival debridement with PEND has been used for the nonsurgical management of periodontitis and presents benefits over conventional subgingival debridement, particularly in terms of the capacity to eliminate calculus [14,15]. A clinical study described that the implementation of PEND presented a statistically significant global enhancement in calculus eliminated through subgingival debridement [14]. An in vitro experiment also indicated that PEND offered further advantages for the elimination of calculus in comparison to conventional subgingival debridement [15]. A comparative study assessed the histologic reaction in patients to the elimination of soft and hard deposits by implementing PEND and described no histologic manifestations of persistent inflammation. Furthermore, bone healing and the development of a long junctional epithelium were additionally perceived in formerly affected root tissues [16]. Furthermore, a previous systematic review evaluated the ability of dentists to remove calculus by PEND, finding superior results to those observed with the implementation of traditional subgingival debridement. However, the properties of PEND in improving clinical parameters were not adequately explored [13]. Unfortunately, that review evaluated some randomized clinical trials with no follow-up and others with a follow-up of a few weeks. Furthermore, most of the trials had very small sample sizes that did not provide enough power to find differences between PEND and subgingival debridement in parameters such as probing depth and bleeding on probing. The randomized clinical trials studied also presented great heterogeneity and a considerable risk of bias. Considering these limitations, it is important to carry out an updated systematic review that allows for obtaining unbiased results.

The objective of this systematic review is to evaluate the clinical efficacy of periodontal endoscopy during subgingival debridement to treat periodontitis. Three widely recognized clinical parameters, such as probing depth, clinical attachment level, and bleeding on probing, were considered.

## 2. Materials and Methods

### 2.1. Protocol Record

This systematic review of randomized clinical trials was performed considering the Preferred Reporting Items for Systematic Reviews and Meta-analyses (PRISMA) guide [17]. Furthermore, its protocol was recorded in PROSPERO (the International Prospective Register of Systematic Reviews—receipt 402788).

### 2.2. Studies Qualification

The research question was addressed as follows:

Population: Patients diagnosed with periodontitis without the presence of systemic diseases.

Intervention: PEND during subgingival debridement.

Comparison: subgingival debridement.

Outcomes: primary, probing depth, and clinical attachment level; secondary, bleeding on probing.

Study design and follow-up: randomized clinical trials with follow-up of at least 6 months.

### 2.3. Selection Criteria

Inclusion criteria: Only randomized clinical trials with a duration of 6 months or more that studied patients diagnosed with periodontitis who were systemically healthy and treated with subgingival debridement and PEND were included. Based on the information from a previous trial that implemented PEND [18], and to ensure sufficient power to establish differences between the comparison groups, only randomized clinical trials with a sample size greater than 30 patients were considered.

Exclusion criteria: Randomized clinical trials with surgical or antimicrobial interventions, in vitro assays, as well as animal studies and duplicate investigations, were excluded.

### 2.4. Search Strategy

Online sources included PubMed, Web of Sciences, SCOPUS, and SCIELO. The gray literature (Google Scholar and OpenGrey) was also revised. MeSH terms and keywords were considered to search randomized clinical trials in all languages until January 2023. The terms included were periodontitis, periodontal diseases, mechanical therapy, subgingival debridement, non-surgical periodontal treatment, periodontal pocket, clinical attachment loss, probing depth, periodontal endoscope, endoscope, endoscopy, and perioscope.

### 2.5. Review Process

Both investigators assessed the titles and abstracts and chose randomized clinical trials to consider the full text for probable admissibility. Disagreements were handled by consensus. However, the statistical test of agreement between examiners gave a very satisfactory result (Cohen’s Kappa > 90).

### 2.6. Data Collection

It was agreed to use a tool to include the most relevant information from the selected randomized clinical trials. This process was carried out individually by the researchers to later compare the information acquired. This information included the data related to the authorship and publication date of the randomized clinical trials, as well as some demographic characteristics of the patients and the results of the interventions, considering the outcome variables studied in this review.

### 2.7. Quality Evaluation

Both authors independently assessed the quality and risk of bias of the included randomized clinical trials, using a widely known tool for this purpose [19].

## 3. Results

**Figure 1:**
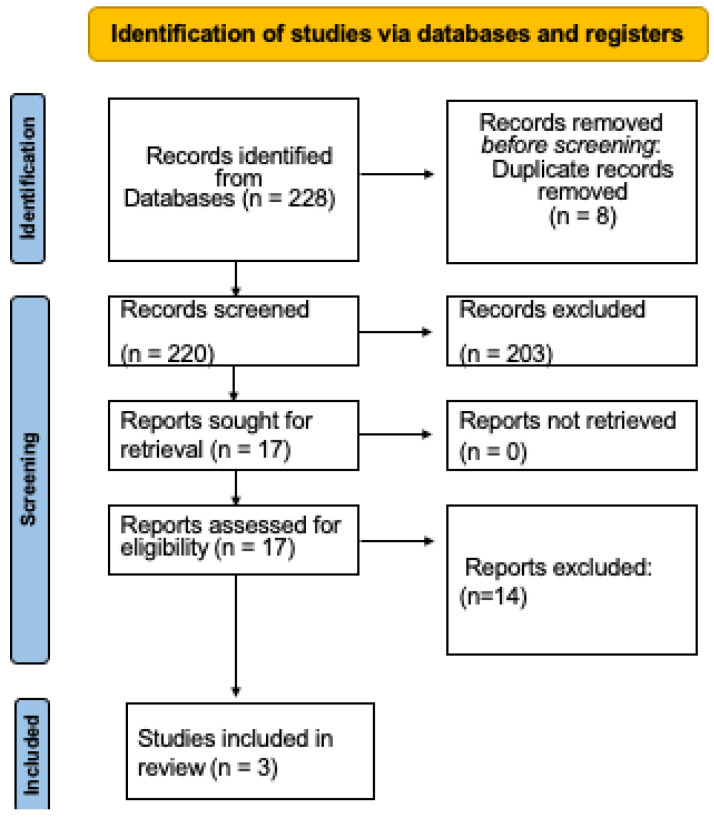
Flowchart of the randomized clinical trial selection process.

The online exploration generated 228 reports. After revising the titles and abstracts, 203 research studies were discounted for their inappropriateness, and 8 duplicate studies were also discarded. After reading the full text, 14 trials were excluded because they did not meet any of the selection criteria. Lastly, three randomized clinical trials were analyzed in this study (Figure 1) [6,20,21]. 

The descriptions of the investigated randomized clinical trials are depicted in Table 1. These parallel design trials were single-blinded and controlled, and they were published in 2020 [21] and 2022 [6,20].

These randomized clinical trials had very similar sample sizes, totaling 113 patients diagnosed with chronic periodontitis. Two randomized clinical trials were followed up for 6 months [20,21], while the remaining one evaluated patients for 12 months [6]. The three randomized clinical trials assessed the three clinical parameters that were part of the outcome variables (probing depth, clinical attachment level, and bleeding on probing) studied in this systematic review. Plaque index was also evaluated in two trials. Moreover, one trial evaluated two additional variables that measured radiographic bone level and change in gingival recession [6]. 

The periodontal therapy performed in each of the studies is described below. Naicker et al. [6] indicated that the patients were treated with root surface debridement using hand and powered instruments. Wu et al. [20] implemented scaling and root planing using an ultrasonic device and hand curettes, while Zhang et al. [21] performed supragingival scaling and traditional scaling and root planing using ultrasonic means and curettes.

All the randomized clinical trials studied found a greater reduction in probing depth in the group intervening with PEND compared to the controls (*p* < 0.05) (Table 1). The interval of preoperative dimensions for the PEND groups it was 4.2–6.1 mm, while for the control groups was 4.6–5.9 mm (*p* > 0.05). Instead, the postoperative values were 2.7–3.1 mm for PEND and 3–3.8 mm for the controls (*p* < 0.05). Consequently, the improvement in probing depth was 2.5 mm for PEND and 1.8 mm for the control groups, respectively (*p* < 0.05). Interestingly, one randomized clinical trial described that the PEND group presented a significantly inferior proportion of probing depths of 7 to 9 mm at 12 months (0.5%) as compared to the control group (1.84%) (*p* = 0.03).

The scenario for the clinical attachment level parameter was different (Table 1). Although two randomized clinical trials found greater improvement in this parameter in the PEND group versus the controls (1.4 mm versus 1.15 mm and 2 mm versus 1.28 mm) [6,20], only the other randomized clinical trial found statistically significant differences after 6 months, with an improvement of 1.73 mm and 1.13 mm for the PEND and control groups, respectively (*p* < 0.001) [21].

Two randomized clinical trials described significant differences in bleeding on probing in favor of PEND, with an average reduction of 43% versus 21% in the control groups [6,18]. Similarly, two randomized clinical trials also presented significant differences in the percentages of bacterial plaque in favor of PEND [6,20]. Naicker et al. [6] described a plaque index of 25.61 ± 3.9% for the PEND group and 30.11 ± 6.3% for the control group (*p* < 0.05), while Wu et al. [20] observed a higher diminution in the PEND group (0.49 ± 0.21 versus 0.72 ± 0.28; *p* = 0.021).

On the other hand, it was also reported in one randomized clinical trial that there was more radiographic bone gain in the PEND group (0.69 ± 0.3 mm) as compared to the control group (0.49 ± 0.2 mm) (*p* = 0.03), including multi-rooted teeth (PEND group = 0.83 ± 0.45 mm versus control group = 0.46 ± 0.36 mm) (*p* = 0.001). This same clinical trial also showed less variation in the dimension of gingival recession in the PEND group (−0.13 ± 0.2 mm) compared to the control group (−0.50 ± 0.6 mm) (*p* = 0.01) [6].

Only one of the randomized clinical trials reviewed had a high risk of bias (Table 2). However, the randomized clinical trials studied in this review had considerable heterogeneity in their designs, visualized at different follow-up times, variability in the moments of the evaluation of the parameters, differences in the form of evaluating the clinical characteristics, variability in the clinical parameters evaluated, among others, aspects that make it difficult to carry out a quantitative assessment.

## 4. Discussion

This systematic review is the first to evaluate the clinical efficacy of PEND to treat periodontitis, including randomized clinical trials with at least 6 months of follow-up. Earlier, a systematic review assessed the advantages of PEND [13]. Nevertheless, its level of evidence is troublesome because its selection criteria were very lax and included clinical trials without follow-up and very small sample sizes, among others, generating biases that require attention.

The relevant eligibility criteria of the current systematic review permitted the study of 3 randomized clinical trials [6,20,21], with follow-up periods between 6 and 12 months and sample sizes greater than 37 patients, while the review carried out by Kuang et al. [13] studied 4 randomized clinical trials that explored the same periodontal parameters considered here, with follow-ups of up to 3 months and sample sizes, for example, of only seven patients to compare two groups. It is important to note that the present review did not include any of the randomized clinical trials studied in the previous review for the reasons previously described.

Calculus has microbial elements that are directly related to the inflammatory reaction that causes the loss of periodontal tissues. Subgingival debridement away from visual contact has been reported to lack specificity, sensitivity, and reproducibility, such that complete removal of subgingival soft and hard deposits is difficult to complete. This limitation increases as the pockets get deeper [22]. On the other hand, while an open periodontal flap may eliminate hard and soft deposits under direct visualization and achieve better debridement efficacy, it may additionally generate postoperative soft tissue involvement, root surface exposure, discomfort, and a lengthier healing period. Moreover, in less healthy patients, surgical therapy is complicated and has a higher possibility of complications, whereas certain patients are afraid to undergo surgical dental therapy [23]. Improving nonsurgical subgingival debridement is therefore important in controlling residual periodontal pocket sites and the overall prognosis of the teeth [20]. In this context, it is important to have alternatives that improve subgingival debridement with the support of new technologies such as PEND.

PEND is a minimally invasive procedure that facilitates visualization of periodontal tissues, which in turn improves the possibility of finding and removing calculus located on the subgingival tooth surface [20]. Stambaugh et al. [24] initially presented the configuration of PEND, inferred endoscopic pictures of the periodontal context, and determined that PEND offered explicit and real-time vision and amplification of the root subgingival area, calculus, and gingiva, which could benefit analysis and treatment by clinicians. Researchers have directed several experimental reports to study the benefits of implementing PEND over usual subgingival debridement. Thus, the ability of subgingival debridement with PEND to remove calculus has been described in several studies [13,14,15,25]. It was observed that the utilization of PEND might surpass the application of an explorer to detect the presence of remnant calculus. Consequently, it is comprehensible that PEND increases the dentist’s capacity to eliminate deposits, and consequently, it has been indicated that PEND justifies being commended for periodontal management [13].

Although the efficacy of PEND during subgingival debridement has been demonstrated [13], there are few randomized clinical trials that compare relevant clinical parameters such as probing depth, clinical attachment level, and bleeding on probing between PEND with subgingival debridement and subgingival debridement alone.

The current systematic review found a statistically significant improvement in the probing depth parameter in favor of PEND, corroborating previous studies [26,27]. Liao et al. [26] observed that in those sites with probing depth ≥6 mm in anterior teeth, the probing depth value in the PEND group after 3 months was significantly lower than that in the subgingival debridement group (3.2 ± 0.9 mm versus 3.7 ± 0.9 mm; *p* < 0.05). In a retrospective study, a decrease in probing depths for all kinds of teeth, predominantly in posterior teeth with deep pockets, was observed. Fifty-five percent of molars with pocket depths commencing at 7 to 9 mm diminished to >5 mm, while 69% of molars with pockets fluctuating from 5 to 6 mm diminished to >4 mm [27]. Beside the four clinical trials studied in the review by Kuang et al. [13] also found improvement in probing depth in patients treated with PEND, but in only one of them, the difference was statistically significant. Kuang et al. [13] also recognized that it is not appropriate to evaluate the therapeutic results of periodontal therapy in the short term because periodontitis is associated with prolonged development. Here, the improvement in probing depth was 2.5 mm and continued to be significant at a 12 month follow-up. Previous studies corroborate that healing and periodontal maturation continue after 9 to 12 months, including improvement in the probing depth parameter [28,29]. A systematic review also described that in deep periodontal pockets, it is difficult to perform subgingival debridement, recommending other treatment alternatives [23]. In this regard, it has been speculated that PEND facilitates the visualization of root surfaces, allowing adequate debridement of deep pockets, thus reducing the need for open flap treatment [6]. In the current review, only one study presented results considering residual pockets. In this regard, Wu et al. [20] indicated that subgingival debridement plus PEND significantly improved probing depth in residual pockets >5 mm (3.12 ± 0.63 vs. 4.0 ± 0.68 mm, *p* = 0.001).

Considering the results related to clinical attachment level, this systematic review also found clinical improvement of this parameter in patients treated with subgingival debridement and PEND, especially in a randomized clinical trial that described statistically significant differences [21]. Similarly, Liao et al. [26] observed that in those sites with probing depth ≥6 mm in anterior teeth, the clinical attachment level tended to be inferior in favor of PEND compared to subgingival debridement (2.9 ± 1.2 mm versus 3.6 ± 1.3 mm; *p* = 0.061). Seeing that PEND can achieve better periodontal debridement and greater removal of residual deposits, supported by the advantages of root visualization, an improvement in the level of clinical attachment is predictable. However, more randomized clinical trials with adequate follow-up periods are required to corroborate these results.

The bleeding on probing and plaque index also presented statistically significant clinical improvement in this systematic review, in favor of the groups intervened with PEND. Regarding these parameters and like that described above, the four randomized clinical trials that evaluated periodontal parameters in the review by Kuang et al. [13] also found improvement in bleeding on probing, but in only one of them, the differences were statistically significant. That study described a significant change in bleeding on probing after 3 months in favor of PEND compared to subgingival debridement (*p* = 0.036). Similarly, mean variations in the gingival index were additionally found to be superior in the PEND group compared to subgingival debridement after 8 weeks (*p* = 0.006) and 3 months (*p* = 0.0001) [27]. This corroborates once again the need to evaluate the periodontal parameters in the longer term, as observed in the studies included in the current review. In sum, the association of bleeding on probing and plaque index with subgingival calculus has been widely recognized [16,30]. In this regard, it has been pointed out that clinicians who use PEND may complete superior debridement and leave fewer subgingival deposits; therefore, it is considered that more diminutions in bleeding on probing and gingival inflammation could be obtained by implementing PEND [13]. This is the relevance of the visualization achieved with the PEND for the removal of residual subgingival calculus.

In this review, improvement was also reported in bone gain in favor of subgingival debridement with PEND. This result was also perceived with multi-rooted teeth [6]. Similar results were reported for infrabony defects using microscopes and magnifying lenses [31]. It has been speculated that greater bone filling may occur using PEND because visualization allows for more effective removal of biofilm and remaining subgingival calculus, generating a greater impact on healing [6].

Herein, an improvement in the dimension of the gingival recession is also described. Like the appreciations already described the advantage in terms of direct visualization offered by PEND can cause less trauma and, in turn, be less tissue invasive in soft tissues. This leads to additional advantages in thin periodontal biotypes and esthetic areas [6].

Interestingly, one study described that after 3 months, high-sensitivity C-reactive protein, tumor necrosis factor-alpha, and leukocyte interleukin 17, in the PEND and subgingival debridement groups were significantly improved (*p* < 0.05), whereas the PEND group improved more significantly than the subgingival debridement group (*p* < 0.05) [32]. These results confirm the additional benefits of PEND in improving inflammatory markers related to periodontitis.

On the other hand, it has also been informed that the outcomes of a quantitative exploration concerning mean therapy times, indicated that performing PEND consumed more time than employed during usual subgingival debridement [13]. Nevertheless, researchers observed that the average management period needed to complete subgingival debridement by means of implementing PEND diminished and approached the interval of time necessary to accomplish conventional subgingival debridement by the dentist who remained familiar with implementing the equipment [14]. Consequently, it was indicated that time consumption must not be a difficulty for the operation and promotion of PEND [13].

Persistent remaining pockets are usually noticed as requiring additional therapy, and this management remains demanding, particularly in terms of removing sticky calcified deposits and toxins [20]. Thus, the findings of the current systematic review indicate that subgingival debridement with PEND shows significant efficacy in the clinical improvement of periodontal parameters such as probing depth, clinical attachment level, and bleeding on probing compared to subgingival debridement alone, suggesting a favorable outcome of this combined management in obtaining periodontal health. Despite these results, this study has some limitations. Important outcomes, such as pocket closure and residual pockets after non-surgical periodontal therapy and PEND, and indications for the sites more difficult for treatment, such as furcation involvement, were not considered in most of the clinical trials evaluated. Moreover, as in the previous review by Kuang et al. [13], the included randomized clinical trials were few. However, in the present review, the included studies had longer follow-ups with larger sample sizes, which allowed us to demonstrate the efficacy of PEND. In any case, better-quality clinical trials are required to corroborate the results found here. On the other hand, the randomized clinical trials studied in this systematic review were heterogeneous, which prevented a more exhaustive analysis. The same difficulties have been reported by other recent systematic reviews [33,34]. This heterogeneous behavior of the studies included in the systematic reviews warrants the standardization of clinical protocols to make comparisons without a bias between the investigations. It is also important to note that all the trials included in this review started before the publication of the current classification of periodontal diagnoses. Therefore, we present them based on the diagnoses indicated in each of the studies.

## 5. Conclusions

This systematic review demonstrated that subgingival debridement with PEND had more efficacy in improving periodontal parameters such as probing depth, clinical attachment level, and bleeding on probing for the treatment of periodontitis compared to subgingival debridement alone. However, more randomized, controlled clinical trials with adequate sample sizes and long follow-up periods are required to corroborate the results found in the current systematic review.

## Figures and Tables

**Table 1:** Descriptions of the clinical trials assessed.

Authors Publication Date	Periodontal Diagnoses	Patients	Mean AGE	Female Male	Intervention Control	Main and Secondary Outcomes	Follow-Up
Naicker et al. 2022 [6]	Moderate to Severe Chronic Periodontitis	38	52 years	24/14	Root surface debridement with perioscope (Test group) or root surface debridement only (Control group).	At 12 months, the test group had a significantly more reduced probing depth (Test group: 2.70 + 0.2 mm; Control group: 2.98 ± 0.4 mm). The test group presented a significantly lower % of probing depth 7 to 9 mm at three (0.72 ± 1.2%) and 12 months (0.5 ± 1.0%) as compared with the control group (2.25 ± 2.9%; 1.84 ± 2.3%) (*p* = 0.03). No differences were detected in the clinical attachment level. At 12 months, the test group had a significantly inferior mean bleeding on probing (Test group: 4.3 ± 3.2%; Control group: 11.95 ± 7.1%).	12 months
Wu et al. 2022 [20]	Moderate to Severe Chronic Periodontitis	37	37 years	22/15	Scaling and root planing plus periodontal endoscopy (Test group) or scaling and root planing alone (control group).	More significant reduction in probing depth were presented in the test group at the 6-month follow-up (3.12 ± 0.63 vs. 4.0 ± 0.68 mm; *p* = 0.001). No significant differences in clinical attachment level or bleeding on probing were perceived.	6 months
Zhang et al. 2020 [21]	Chronic periodontitis	38	36 years	24/14	Scaling and root planing plus periodontal endoscopy (Test group) or scaling and root planing alone (control group).	Compared with those in the scaling and root planing group, probing depth at 3 and 6 months after therapy, and clinical attachment level and bleeding on probing at 6 months after treatment were reduced in the endoscope group (*p* < 0.05).	6 months

**Table 2:** Critical appraisal of the studied randomized clinical trials.

Randomized Clinical Trial	Randomization	Blinding	Withdraw	Appropriate Randomization	Appropriate Blinding	Total
Naicker et al. 2022 [6]	1	0	1	0	0	2
Wu et al. 2022 [20]	1	1	1	1	0	4
Zhang et al.2020 [21]	1	1	1	0	0	3

## Data Availability

The data obtained in this review were pooled from the included investigations.
